# Intestinal-Type Mucinous Borderline Ovarian Tumour in Pregnancy With a Non-invasive Omental Mucinous Deposit: A Case Report

**DOI:** 10.7759/cureus.113626

**Published:** 2026-07-29

**Authors:** Nursyafizan Amalina Rahamzan, Norsollehin Adnan

**Affiliations:** 1 Obstetrics and Gynaecology, Shah Alam Hospital, Shah Alam, MYS

**Keywords:** conservative cystectomy, expectant management, maternal-fetal outcomes, mucinous borderline ovarian tumor, pregnancy

## Abstract

Borderline ovarian tumours during pregnancy are uncommon and require a balance between maternal oncological safety, fetal well-being, and fertility preservation. Intestinal-type mucinous borderline ovarian tumours associated with a non-invasive omental mucinous deposit are particularly unusual.

A 31-year-old primigravida was found to have an asymptomatic 9.8 cm adnexal mass at 11 weeks’ gestation. Serial ultrasonography showed no papillary projections, solid components, ascites or abnormal Doppler flow; however, the mass progressively enlarged to 15 cm by 36 weeks. At 37 weeks, induction of labour for uncontrolled hypertension was complicated by fetal distress, necessitating emergency caesarean delivery. Intraoperative examination revealed a 15 cm thin-walled, biloculated left ovarian cyst without grossly suspicious features. Fertility-sparing cystectomy and ovarian reconstruction were performed, during which inadvertent cyst rupture occurred. The macroscopically normal omentum was biopsied. Histopathological examination confirmed an intestinal-type mucinous borderline ovarian tumour. Immunohistochemistry showed diffuse cytokeratin 7 (CK7)* *positivity, focal CK20 and Caudal-type homeobox transcription factor (CDX2)positivity, and negative oestrogen receptor​​​​​​​ (ER)and paired-box family of transcription factor​​​​​​​ (PAX8) staining, supporting a primary ovarian tumour with intestinal-type differentiation. The omental specimen contained a non-invasive mucinous epithelial deposit without evidence of malignancy, considered most likely related to surgical spillage. Following counselling regarding the risks of recurrence and progression, the patient elected conservative surveillance and remained clinically and radiologically free of recurrence during short-term postpartum follow-up and is planned for long-term surveillance 3 monthly follow up for 2 years, then 6-monthly up to at least 5 years. If fertility is no longer desired, completion surgery will be discussed.

This case demonstrates that reassuring sonographic and intraoperative morphology does not exclude a borderline ovarian tumour when an adnexal mass progressively enlarges. Fertility-sparing management may be appropriate in carefully selected patients, provided that recurrence risk and the need for long-term surveillance are clearly addressed.

## Introduction

Borderline ovarian tumours (BOTs) represent a significant subset of ovarian tumours, accounting for 10%-20% of all epithelial-origin cases. Serous and mucinous subtypes represent the two primary histological classifications of borderline ovarian tumours [1].

Although systematic screening for adnexal masses is now standard in the first trimester, and despite the increasing age of pregnant women, the diagnosis of a borderline ovarian tumour during pregnancy remains rare [2]. BOTs occur in approximately 1.8 to 4.8 of 100,000 women per year [3]. The antenatal diagnosis of BOTs remains a significant challenge because no single ultrasound feature is pathognomonic, although multi-septation and papillary projections are recognised preoperative sonographic features [4].

A multi-centre study reported that mucinous BOTs were more common than serous BOTs in pregnant patients (48% vs. 30%) [5]. Pregnant women also demonstrated a higher prevalence of advanced-stage disease and aggressive histological features compared with the non-pregnant population [5]. These findings may be related to the hormonal changes of pregnancy, as elevated oestrogen and progesterone levels can stimulate the hormone receptors frequently expressed in BOTs, potentially influencing tumour growth and progression [2].

Despite being rare, these tumours require a management strategy that balances clinical efficacy to reduce recurrence risk with the conservation of future fertility [2]. Current practice varies, and a lack of robust comparative data prevents the formulation of a specific consensus on whether cystectomy or oophorectomy is the superior surgical approach in all young patients. European Society of Gynaecological Oncology (ESGO) recommended that in the case of unilateral serous and seromucinous borderline ovarian tumour, options of unilateral salpingo-oophorectomy or cystectomy with macroscopic healthy ovarian tissue sparing are both acceptable. Patients of the latter, however, should be counseled about up to a 30% risk of local and ovarian recurrence - this doesn't affect the overall survival, but yields better fertility outcomes [6]. The execution and degree of staging procedure during surgery also vary from clinician to clinician, especially in cases where no obvious abnormalities are seen during inspection of the abdominal cavity [7].

We report a case of a primigravida with an enlarging ovarian cyst with benign features detected early in pregnancy that was managed conservatively until delivery, with a subsequent intraoperative cystectomy at caesarean section. Histopathology revealed a mucinous borderline ovarian tumour with non-invasive omental implants. This case highlights the limitations of ultrasonography as a modality for antenatal imaging, the complexity of managing adnexal masses during pregnancy, and the importance of individualised, fertility-sparing decision-making in young patients.

## Case presentation

A 31-year-old Malay lady in her first pregnancy was referred from a health clinic at 11 weeks and 3 days of gestation for evaluation for an adnexal mass detected during her booking. She was asymptomatic. She had a background of chronic hypertension. Otherwise, she had no personal history of benign ovarian cysts or pelvic inflammatory disease. No history of benign ovarian tumour, lung, bone, or pancreatic cancer, or leukaemia runs in her family. She neither smoked nor consumed alcohol. Her antenatal booking blood investigations were normal. Her booking BMI was 25 kg/m². Her booking blood pressure was 152/108mmHg.

Initial transabdominal ultrasound demonstrated a single viable fetus that corresponded to the gestational age. There was a right ovarian cyst measuring 9.8 cm x 5.8 cm. The cyst was described as uniloculated with no papillary projection, no solid area, and no free fluid, and the colour Doppler showed no abnormal flow. The contralateral ovary was normal.

A repeated ultrasound at 14 weeks revealed a reduction of the cyst size to 6.7 cm x 6.6 cm with no new suspicious features. Tumour markers were not requested antenatally. Surgical management was discussed with the patient. However, in view of no symptoms and reassuring sonographic features, she opted for conservative management. 

The subsequent follow-up ultrasound at 25 weeks and 1 day revealed interval enlargement of the cyst to 9.4 cm x 8.1 cm and a further increase to 15 cm x 8 cm at 36 weeks and 2 days. The sonographic features remained benign, and the patient remained asymptomatic. Fetal growth was normal and appropriate for age.

At 37 weeks and 3 days, she was admitted for uncontrolled hypertension that required induction of labour. The labour was complicated by fetal distress with pathological cardiotocograph (CTG). CTG noted a baseline heart rate of 125, poor beat-to-beat variability, persistent early deceleration for more than 50 minutes with no shouldering effect - no improvement despite conservative methods - necessitating a caesarean section.

A Pfannenstiel incision was made. Upon entering the peritoneal cavity, no ascites was observed. A live male infant weighing 2,330 g was delivered. The Apgar scores were 9 and 10 at 1 and 5 minutes, respectively. Umbilical arterial blood gas analysis showed a pH of 7.22, bicarbonate concentration of 21.7 mmol/L, and a base excess of −6.3 mmol/L.

**Figure 1:**
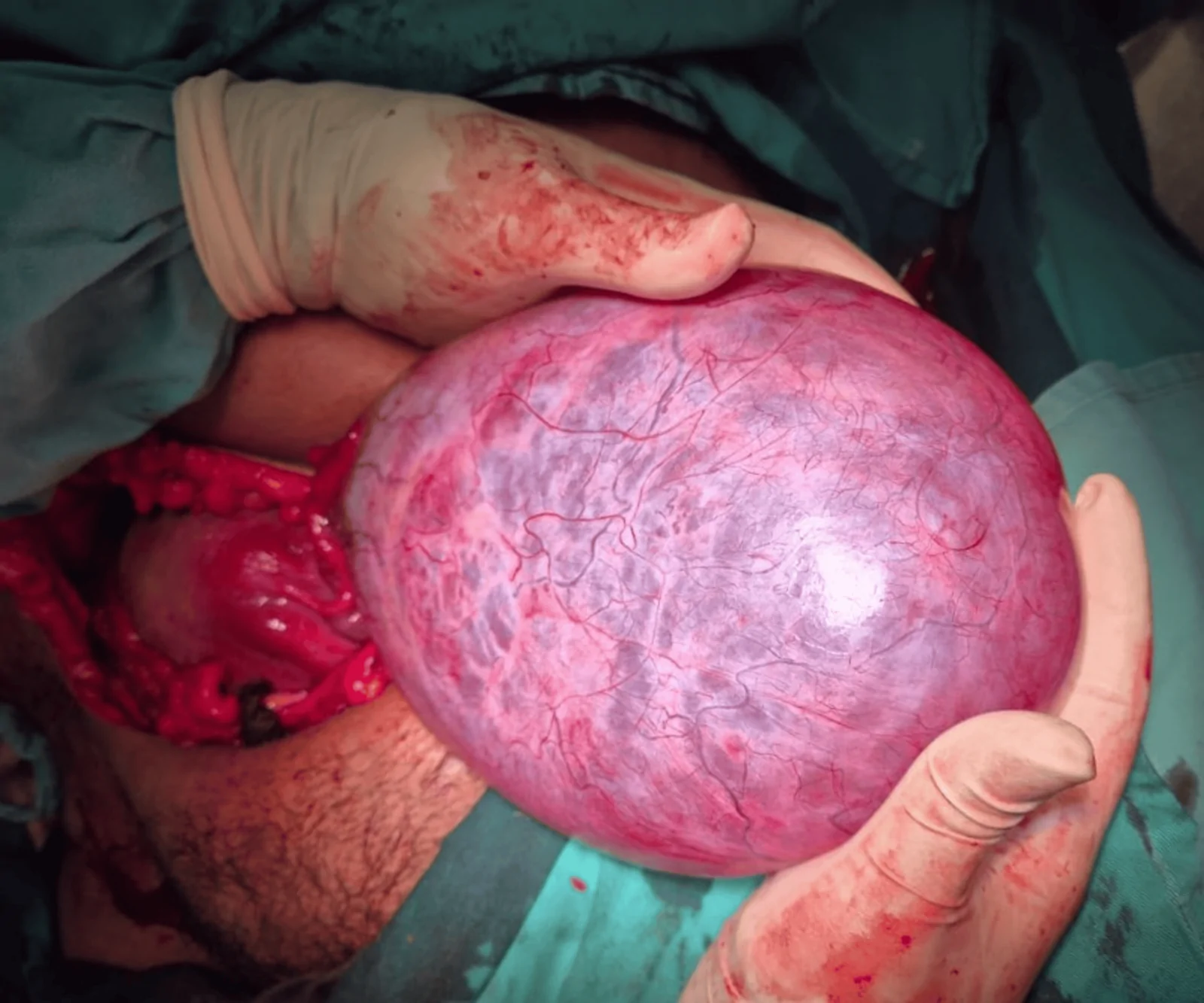
Left ovarian mass (15 cm × 15 cm) exteriorized intraoperatively during caesarean section.

Once the uterus was closed and the haemostasis confirmed, the peritoneal cavity was explored. A left ovarian cyst measuring 15 cm x 12 cm was visualised. The cyst was biloculated with a thin wall, with no abnormal vessels or solid components. The cyst was exteriorised (Figure 1), and a cystectomy was performed. The cyst ruptured during cystectomy, revealing serous and mucinous contents. Subsequently, the left ovary was reconstructed using 3-0 Prolene. The right ovary, bilateral fallopian tubes, uterus, bowel, liver surface, and appendix were all normal. Macroscopically, the omentum appeared normal, and a biopsy was taken. The surgery lasted 1 hour and 44 minutes, with an estimated blood loss of 600 mL.

The discrepancy in laterality between antenatal imaging and intraoperative findings was attributed to sonographic mislabelling in pregnancy, which has been reported in large adnexal masses. The patient was discharged well on postoperative day 3. At one month postpartum, she attended the outpatient obstetrics and gynaecology clinic to review her blood pressure control and histopathological results.

**Figure 2:**
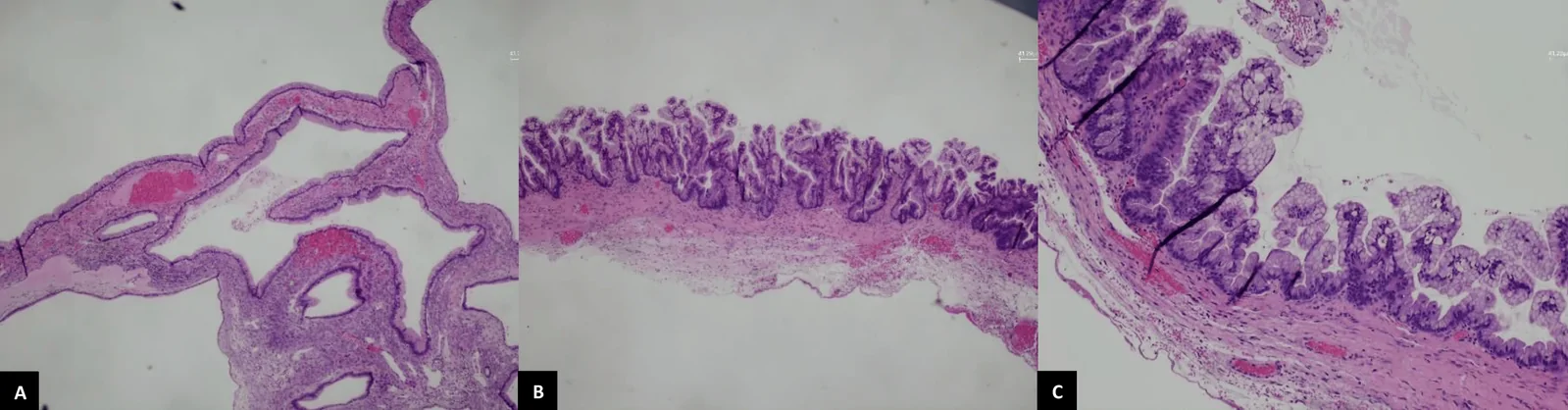
Pathology images of the tumors with Hematoxylin-Eosin staining. (A) Area of mucinous cystadenoma. Fibro-ovarian cyst wall lined by a single layer of gastrointestinal-type mucinous columnar epithelium (original magnification ×4); (B) Areas of epithelial proliferation with more complex architecture, tufting, and villous formation (original magnification ×4); (C) The cells show mild atypia with reduced cytoplasmic mucin, nuclear stratification, hyperchromatic with some loss of polarity (original magnification ×10).

**Figure 3:**
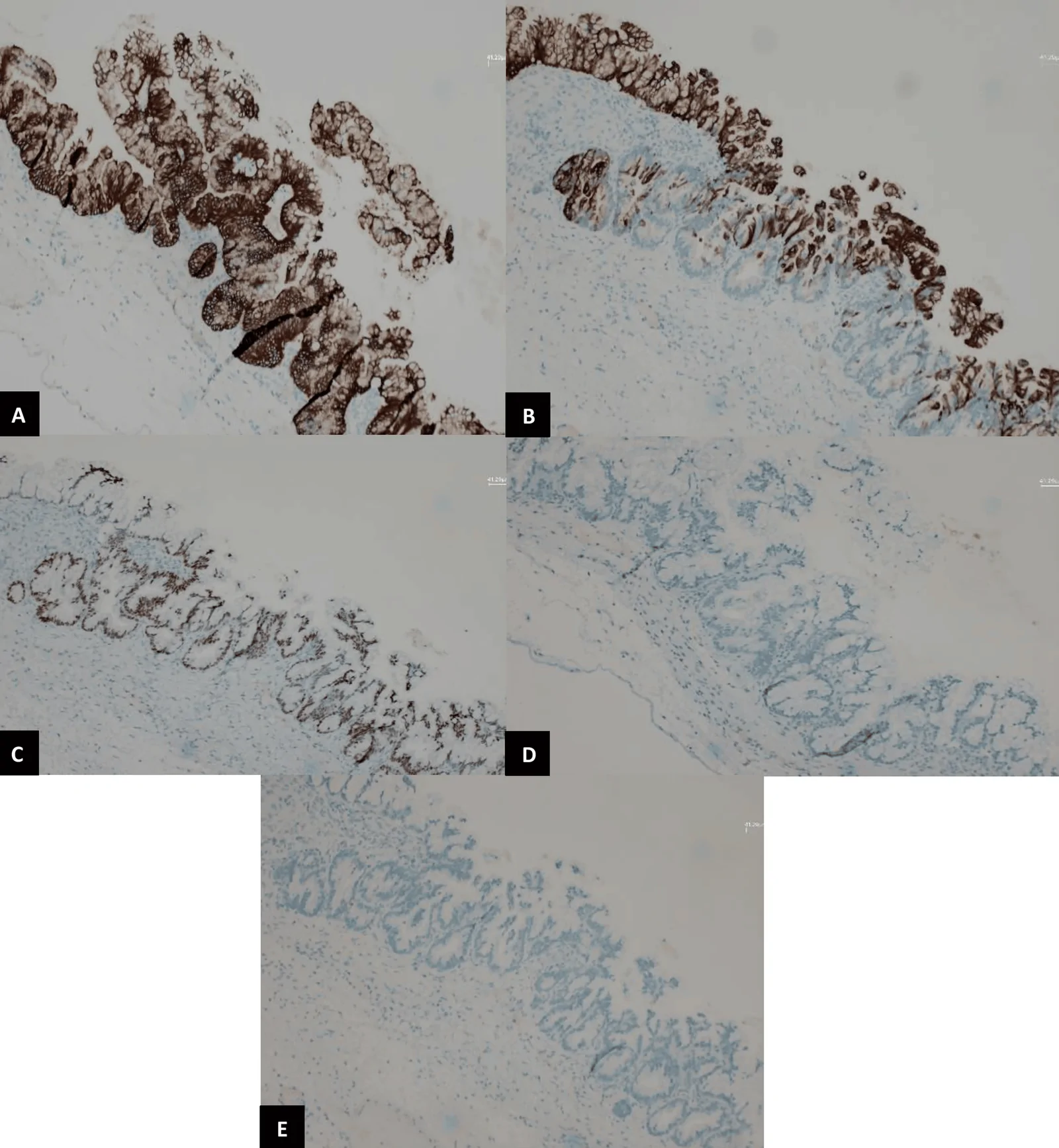
Immunohistochemistry panel of the tumour (original magnification ×10). (A) CK7 is diffusely positive, supporting a primary gynaecological origin; (B) CK20 is focally positive and (C) CDX2 is positive, confirming intestinal-type differentiation; (D) ER is negative, excluding endometrioid carcinoma with mucinous differentiation; and (E) PAX8 is negative, excluding other Müllerian epithelial tumours. The immunohistochemistry panel demonstrates intestinal-type differentiation of primary ovarian origin. CK: cytokeratin; CDX: caudal-type homeobox transcription factor; ER: oestrogen receptor; PAX8: paired-box family of transcription factor

The histopathological report described the ovarian cyst wall as a mucinous borderline ovarian tumour (Figure 2). Cytokeratin 7 (CK7) was positive (Figure 3A), CK20 was focal positive (Figure 3B), caudal-type homeobox transcription factor (CDX2) was focal positive (Figure 3C), oestrogen receptor (ER) was negative (Figure 3D), and paired-box family of transcription factor (PAX8) was negative (Figure 3E) on the immunohistochemistry results. The omentum was reported as a non-invasive mucinous epithelial implant/surface most likely related to surgical spillage, and no malignancy was seen. All the features are consistent with primary ovarian origin with intestinal-type differentiation.

Borderline ovarian tumours are staged using the International Federation of Gynecology and Obstetrics (FIGO) staging system for ovarian, fallopian tube and peritoneal tumours. The intraoperative cyst rupture would have been classified as FIGO stage IC1 because of surgical spill. Although FIGO stage may not necessarily alter the need for adjuvant treatment following complete resection of a borderline ovarian tumour, it remains important for prognostication, patient counselling and surveillance planning.

Given the patient’s young age and primigravida status, individualised counselling was provided regarding the risks and benefits of completion salpingo-oophorectomy versus fertility-sparing surveillance. She chose conservative management with close follow-up.

At 4 months postpartum, the patient remains asymptomatic with no clinical or radiological evidence of recurrence. Tumour marker Ca125 sent during our surveillance follow-up was normal (2.7 U/mL). The case was discussed with a gynaecologist, and the patient is planned for 3-monthly follow-up for 2 years, then 6-monthly up to at least 5 years. If fertility is no longer desired, completion surgery will be discussed.

## Discussion

The management of a large adnexal mass during pregnancy presents a significant clinical dilemma, balancing maternal oncological safety with foetal well-being. This case of mucinous borderline ovarian tumour highlights several practical learning points: the limitations of antenatal imaging, the restricted role of biochemical markers in pregnancy, and the validity of individualised conservative surgery when fertility preservation is a major concern.

A unique feature of this case was the discrepancy in laterality between antenatal imaging and the intraoperative finding. As pregnancy progresses, enlargement of the gravid uterus may significantly distort normal pelvic anatomy, resulting in superior or contralateral displacement of the ovaries and adnexa, particularly in the presence of a large adnexal mass, thereby complicating both imaging interpretation and surgical localisation [8]. This supports the need for deliberate bilateral peritoneal and adnexal assessment at surgery, even when the preoperative scan assigns the mass to one side. 

Tumour markers were not requested antenatally because their interpretation is less specific in pregnancy. CA 125 concentrations are physiologically elevated during the first trimester of pregnancy, further limiting its diagnostic value as a tumour marker in pregnant women. Similarly, germ-cell tumour markers such as alpha-fetoprotein, lactate dehydrogenase, and human chorionic gonadotrophin may be naturally elevated during pregnancy, reducing their reliability for distinguishing physiological changes from malignancy. Nonetheless, alternative biomarkers such as inhibin B, anti-Müllerian hormone, human epididymis protein 4 and CA 19-9 may be considered in future similar cases, as these markers are not generally expected to rise physiologically during pregnancy and may provide additional diagnostic support [8].

Significant ultrasonographic features were looked for as per Ultrasonography score (U) in Risk of Malignancy Index (RMI) [9]. An exact RMI score could not be calculated because serum CA-125 was not measured. In this patient, the management strategy therefore appropriately prioritised morphology, symptoms, interval growth, and operative findings over isolated biochemical values.

The role of MRI could have been a useful supplementary modality to further characterise large adnexal or pelvic masses in pregnancy, as in this patient's case. It is also useful in evaluating gastrointestinal conditions such as appendicitis and Crohn's disease, as well as tubo-ovarian abscesses, haemorrhagic degeneration of fibroids, and ovarian torsion. When malignancy is confirmed or strongly suspected, MRI can help determine the extent of disease during pregnancy. T2-weighted and diffusion-weighted sequences are particularly valuable for assessing fluid, inflammation, abscess formation, and pelvic tumours without gadolinium-based contrast, which is generally avoided because of potential fetal safety concerns [10].

The decision to observe the mass antenatally was guided by reassuring sonographic morphology. Although the cyst enlarged to 15 cm, it remained cystic, uniloculated, without papillary projections, solid areas, ascites, or abnormal Doppler flow. Current consensus supports surgical intervention for adnexal masses that persist beyond the first trimester, particularly when they exceed 10 cm or demonstrate sonographic features suspicious for malignancy [10]. For improvement, and to be on the safe side, a multidisciplinary approach, including referral to gynaecologic oncology [11], should be used for masses with rapidly increasing size despite a lack of complex features to better gauge the risk of malignancy.

During the emergency caesarean section for fetal distress, frozen section was not performed. This is a recognised practical limitation in emergency or out-of-hours surgery for many hospitals. In addition, frozen section has known diagnostic limitations in mucinous borderline tumours because of their large size and potential histological heterogeneity [12]. In this case, the smooth thin cyst wall, absence of solid components, absence of abnormal surface vascularity, and normal macroscopic survey supported cystectomy rather than immediate oophorectomy. Inspection of all peritoneal surfaces revealed no abnormal findings. Pelvic and retroperitoneal lymph-node dissection was not performed, as systematic lymphadenectomy has not been considered a routine component of staging for borderline ovarian tumours since 1995 [5].

It is important to distinguish mucinous borderline ovarian tumour (MBOT) from serous borderline ovarian tumour because their clinical and pathological patterns differ. MBOTs are commonly larger and unilateral, whereas serous borderline tumours are more often associated with bilaterality, papillary architecture, and peritoneal disease [11]. This distinction is relevant when interpreting this patient’s large unilateral tumour and planning surveillance.

Immunohistochemical assessment is recommended in all cases of MBOT to confirm an ovarian primary origin, as gastrointestinal metastases may closely resemble MBOTs on morphology [13]. The pathological subtype is also relevant. Intestinal-type MBOTs are characterised by mucin-producing epithelium and may show a spectrum from benign mucinous cystadenoma to borderline proliferation and, rarely, invasive mucinous carcinoma [14]. Recognition of this subtype helps guide counselling about prognosis, recurrence risk, and the need for long-term follow-up.

Postoperative immunohistochemistry supported a primary ovarian mucinous tumour rather than metastatic gastrointestinal disease. The CK7-positive profile, with focal CK20 and CDX2 expression, is compatible with intestinal-type ovarian mucinous differentiation [15]. Immunohistochemistry should be interpreted with the morphology and operative findings, and broader marker panels may provide adjunctive pathological stratification when the diagnosis is uncertain.

Fertility preservation was a central consideration because the patient was young and primigravid. In appropriately selected patients with early-stage borderline ovarian tumours, fertility-sparing surgery is an accepted option, provided that thorough surgical staging is done and the patient understands the need for surveillance and the possibility of recurrence or further surgery if disease recurs [13].

Accidental cyst rupture was an important intraoperative event. Although rupture or spillage may increase concern for recurrence in MBOT [13], the absence of gross malignancy and the non-invasive nature of the omental finding support close surveillance rather than automatic completion surgery.

At 6 months postpartum, the patient remained asymptomatic with no clinical or radiological evidence of recurrence. This supports the initial conservative approach but does not remove the need for long-term follow-up. MBOT recurrences may occur late, even beyond 5 years [13], and rare reports of recurrent or progressive disease reinforce that short-term postpartum reassurance is not equivalent to cure [16,17]. This patient is planned for 3-monthly follow-up for 2 years, then 6-monthly up to at least 5 years. If fertility is no longer desired, completion surgery will be discussed.

## Conclusions

This case demonstrates that benign-appearing sonographic morphology does not exclude a borderline ovarian tumour when an adnexal mass enlarges progressively during pregnancy. Its distinctive features were the unexpected diagnosis of an intestinal-type mucinous borderline ovarian tumour after fertility-sparing cystectomy at emergency caesarean section and the identification of a non-invasive omental mucinous epithelial deposit attributed to intraoperative spillage. Progressive growth should prompt multidisciplinary reassessment despite reassuring morphology. In carefully selected patients, fertility-sparing management may achieve a favourable short-term outcome, provided that the possibility of recurrence and the need for long-term surveillance are clearly addressed.
